# Resilience during uncertainty? Greater social connectedness during COVID‐19 lockdown is associated with reduced distress and fatigue

**DOI:** 10.1111/bjhp.12485

**Published:** 2020-10-25

**Authors:** Jonas P. Nitschke, Paul A. G. Forbes, Nida Ali, Jo Cutler, Matthew A. J. Apps, Patricia L. Lockwood, Claus Lamm

**Affiliations:** ^1^ Faculty of Psychology, Department of Cognition, Emotion, and Methods in Psychology University of Vienna Vienna Austria; ^2^ Department of Psychology McGill University Montreal Quebec Canada; ^3^ Faculty of Psychology, Department of Clinical and Health Psychology University of Vienna Vienna Austria; ^4^ Department of Experimental Psychology University of Oxford Oxford UK; ^5^ Centre for Human Brain Health, School of Psychology University of Birmingham Birmingham UK

**Keywords:** COVID‐19, social connections, resilience, worry, stress, fatigue

## Abstract

**Background:**

Social connections are crucial for our health and well‐being. This is especially true during times of high uncertainty and distress, such as during the COVID‐19 lockdown. This period was characterized by unprecedented physical distancing (often communicated as social distancing) measures resulting in significant changes to people’s usual social lives. Given the potential effects of this disruption on people’s well‐being, it is crucial to identify factors which are associated with negative health outcomes, and conversely, those that promote resilience during times of adversity.

**Aims:**

We examined the relationship between individuals’ levels of social connectedness during lockdown and self‐reported stress, worry, and fatigue. Method: Survey data were collected from 981 individuals in a representative sample of Austrian citizens. Data collection occurred during the last week of a six‐week nationwide lockdown due to the COVID‐19 pandemic. The final sample consisted of 902 participants. Participants were asked to complete validated questionnaires to assess levels of social connectedness as well as measures of perceived stress, worry—both general and COVID‐19 specific—and symptoms of fatigue during the previous two weeks.

**Results:**

Our results demonstrate that greater social connectedness during the lockdown period was associated with lower levels of perceived stress, as well as general and COVID‐19‐specific worries. Furthermore, we found a negative relationship between fatigue and social connectedness, which was mediated by feelings of stress, general worries, and COVID‐19‐specific worries—respectively, indicating that individuals with smaller network sizes, who were highly distressed during the pandemic, were also likely to report feeling more fatigued.

**Conclusion:**

Our findings highlight the important role that social connections play in promoting resilience by buffering against negative physical and mental health outcomes, particularly in times of adversity in times of adversity.

**Statement of contribution**

***What is already known on this subject?***

● The availability of social connections has previously been associated with increased health and well‐being.
● The experience of uncertainty is associated with higher levels of stress and worries.
● Social isolation and loneliness have been associated with negative emotional and physical outcomes.

***What does this study add?***

● The current study provides evidence that being socially connected during a global pandemic is associated with lower levels of distress and symptoms of fatigue.
● In particular, larger and more diverse social networks (i.e., communicating with more individuals) over a two‐week period of physical distancing were associated with lower levels of perceived stress, worries, and fatigue during that time‐period.
● Social connections can potentially buffer against negative physical and mental health outcomes, and promote resilience.

## Background

Humans are social animals, and we rely on each other for our health and well‐being (Snyder‐Mackler et al., 2020). As such, social disconnect can have serious consequences for our physical and mental health (Bzdok & Dunbar, 2020; Holt‐Lunstad, 2018). This is especially true during times of uncertainty and distress when social contact can act as a buffer against adversity and suffering. The COVID‐19 pandemic is having an unprecedented impact on people around the world and can be viewed as a global stressor induced by a threat to health, economic consequences, and a disruption of daily routines. Social connections provide us with support when dealing with negative emotions, such as feelings of distress and worry, especially in such times of adversity and uncertainty ( Cohen & Syme, 1985; Zaki & Williams, 2013). However, to limit the spread of the virus, most countries have instituted varying degrees of social distancing measures (and in particular physical distancing measures), some that require large swaths of the population to stay home and restrict physical proximity to others. In Austria, nationwide lockdown and physical distancing measures were imposed between 14 March 2020 and 30 April 2020. Within a very short period of time, the population was required to largely withdraw from their normal lives and practice physical distancing,^1^ while the social‐, financial‐, and health‐related consequences of COVID‐19 were becoming rapidly apparent. This could increase the potential for negative emotional, physical, and mental health consequences (Cacioppo & Cacioppo, 2014; Christiansen, Larsen, & Lasgaard, 2016; Cohen, Doyle, Skoner, Rabin, & Gwaltney, 1997; Crittenden et al., 2014; Helgeson & Cohen, 1996; Holt‐Lunstad, Smith, & Layton, 2010; Sneed, Cohen, Turner, & Doyle, 2012). Furthermore, physical distancing was coupled with feelings of distress and uncertainty for many individuals (Rajkumar, 2020). Previous research has shown that social isolation, chronic levels of stress, and prolonged feelings of distress and worry can lead to sustained arousal and result in increased levels of fatigue and related somatic complaints (Cho et al., 2019; Nater, Maloney, Heim, & Reeves, 2011; Shevlin et al., 2020; Wyller, Eriksen, & Malterud, 2009). While the experience of distress is a normal reaction to uncertainty (Dickerson & Kemeny, 2004; Vinkers et al., 2020), when unchecked (or unregulated), the severity of the associated negative emotions can be detrimental to mental and physical health (Juster, McEwen, & Lupien, 2010; Wyller et al., 2009). Thus, given the potentially significant downstream social and psychological effects of these lockdown measures, it is crucial to identify factors that might be associated with increased risk for negative health outcomes, and conversely, those that promote resilience during times of adversity (Vinkers et al., 2020).

The emergence of COVID‐19 and the resulting lockdown led to disruptions in people’s typical social lives. This included reductions in face‐to‐face meetings and limited physical contact with social support networks, which would normally help individuals deal with adverse events, such as those presented by the lockdown. An important question is whether social connections can still contribute to resilience despite the distancing measures—a time when social connections are needed the most in order to deal with the disruption due to the pandemic. To this end, we aimed to investigate how perceived levels of stress, generalized worry, COVID‐19‐specific worries, and fatigue were associated with levels of social connectedness in a representative community sample of Austrians when lockdown measures were in place. Here, we operationalize social connectedness as the number of unique individuals that participants communicated with, in a specific time frame during the lockdown. We predicted that being socially connected with others during lockdown would be associated with reduced levels of distress and fatigue in general, as well as a reduction in specific worries related to the COVID‐19 pandemic.

## Methods

### Sample

Using an online panel (recruited by a company specialized in election‐forecasting; Kieskompas BV, Amsterdam, NL) we obtained a representative community sample of 981 Austrians over the age of 18. Of the participants included in the final sample 61% (*n* = 551) had obtained a degree in higher education (vocational school, bachelor, or above). The sample was representative for the variables age and gender. Of the 981 participants, 37 failed to correctly answer the attention check (“Please choose the option 'maybe'”) towards the end of the survey, an additional 42 participants dropped out of the study before reaching the attention check. We proceeded with a final sample of 902 Austrians between 18 and 90 years of age (mean age = 49.60; ±14.45; median = 52). 502 respondents were women (55.6% of sample).^2^ Four participants reported that they had been diagnosed with COVID‐19 (0.44% of sample), and 169 (18.3% of sample) reported that they personally knew someone who was diagnosed.

Data collection occurred from 23 April to 30 April 2020 during a nationwide lockdown due to the COVID‐19 pandemic, when active stay‐at‐home measures had been in place for approximately 5 weeks.^1^ The end of the data collection period was determined by the end of stay‐at‐home orders (on 1 May 2020). All measures were administered in German.

### Social network index

In order to obtain a measure of social connectedness, we used the Social Network Index (SNI; Cohen et al., 1997). The SNI is a self‐report measure which asks participants to provide information about the type and number of social connections they regularly engage in. For this study, participants were asked to specifically respond to each question with reference to their experience in the previous two weeks in lockdown. The SNI classifies 12 social domains, ranging from close familial relationships (i.e., spouse, children, parents, in‐laws, family members), to more distant relationships (i.e., friends, colleagues, employees, classmates, fellow volunteers, religious, and non‐religious group members). From this, an index of social network size (SNI size) can be determined—the total number of people with whom the respondent has regular contact with (in our case, during the last two weeks of the lockdown) (cf. Cohen et al., 1997; Sneed et al., 2012).

In addition, participants were asked to estimate on a 7‐point Likert scale (ranging from 1 = 'not at all' to 7 = 'more than 10 times a day') how often they interacted with their relatives and friends in person, and how often they interacted with them online, in the last 7 days.

### Stress and worry measures

We assessed levels of perceived stress using the Perceived Stress Scale (PSS; Cohen, Kamarck & Mermelstein 1994). Using a 5‐point Likert scale (0 = 'never'; 4 = 'very often') the 10 items of the PSS ask participants to rate how often they experienced specific stressors, or thought about stressful events in the last two weeks (i.e., the same lockdown period as the SNI). A sample item includes 'in the last two weeks, how often have you felt that you were unable to control the important things in your life?'. All items are summed‐up to a stress score (range: 0–40).

To measure generalized levels of worry, we used the Penn State Worry Questionnaire (PSWQ; Meyer, Miller, Metzger, & Borkovec, 1990). The PSWQ asks participants to rate, on a 5‐point Likert scale (1 = 'not typical at all'; 5 = 'very typical'), how typical the listed characteristics are of them. An example item is, 'Many situations make me worry'. All questions were asked in the context of the lockdown measures ('in the last two weeks, during lockdown, how typical or characteristic was each item for you?'). A worry score can be derived by summing all items (range: 16–80).

### Fatigue

To measure levels of fatigue, we used the Chalder Fatigue Questionnaire (CFQ; Chalder et al., 1993). The CFQ is a 11‐item questionnaire assessing levels of fatigue over the past 4 weeks on a 4‐point Likert scale ranging from 0 ('less than usual') to 3 ('much more than usual'). The CFQ measures both somatic symptoms (e.g., 'Do you feel weak?', 'Do you feel sleepy or drowsy?') and the impact of fatigue on daily living (e.g., 'Do you have problems starting things?', 'Do you find it more difficult to find the right word?'). All items are summed for an overall fatigue score (range: 0–33).

### COVID‐19‐specific worries

We asked participants the following six questions about COVID‐19‐specific worries: 'how worried are you about going to the supermarket', 'how worried are you that COVID‐19 will bring lasting changes', 'how worried are you about getting too close to other people', 'how worried are you about being socially isolated', 'how worried are you that you will not see people that are important to you again', and 'how worried are you about losing your job'. Responses ranged from 0 (not worried at all) to 10 (very much worried). The responses for all six items were averaged to a mean‐aggregated COVID‐19‐specific worry score. The responses for all six items were summed to a mean‐aggregated COVID‐19‐specific worry score. Importantly, these questions were not piloted and represent an ad‐hoc inclusion for this specific investigation. In order to test the reliability, we calculated Cronbach’s α for the 6 items used (Taber, 2018). Results indicate an acceptable reliability, α = 0.74. Lastly, we asked participants to provide us with a percentage rating of their perceived risk of contracting COVID‐19 themselves within the next year (risk perception).

### Analyses

To test the association between levels of distress (i.e., PSS, PSWQ, COVID‐19 specific worries) and number of social connections, we ran three separate multivariate regressions. Levels of distress (sum scores for each scale: PSS, PSWQ, COVID‐19‐specific worries) were entered as dependent variables. Since our study sample was stratified by age and gender to provide us with a representative Austrian sample, we included both variables in all analyses. We also included a variable indicating participants’ personal experience with COVID‐19 in all analyses (0 = know of no one diagnosed with COVID‐19; 1 = know of someone, including self, diagnosed with COVID‐19), and a variable indicating levels of COVID‐19‐related financial worries.^3^ Next we ran a multiple regression predicting perceived risk of contracting COVID‐19 in the next year, with the independent variables mentioned above (SNI size, gender, age, financial worries, and COVID experience). Finally, we ran a multiple regression predicting levels of fatigue with SNI size. We again included gender, age, COVID experience, and financial worries as covariates. As perceived levels of stress are often associated with somatic symptoms such as fatigue (Nater et al., 2011; Strahler, Skoluda, Rohleder, & Nater, 2016), and recent research indicates a direct relationship between perceived COVID‐19‐specific worries and fatigue (Shevlin et al., 2020) we ran mediation analyses using the ‘mediation’ package (Tingley et al., 2014) to test whether COVID‐19‐specific worries mediated the association between SNI size and fatigue. All confidence intervals were bootstrapped. All analyses were conducted in R (version: 3.6.3; R Core Team, 2020).

## Results

### Social connectedness

Participants had, on average, an active (i.e., had contact with, in the last two weeks) social network size of 16.92 people (range: 0 to 41; median = 17) across an average of 6 different domains (e.g., family, friends, and work). As expected, due to lockdown, the mean frequency (1 = 'not at all'; 7 = 'more than 10 times a day') was significantly lower for in‐person interactions (2.51; SD ± 1.26) compared with online interactions (3.18; SD ± 1.07; *t* (901) = −13.401, *p *< .001).

### Levels of distress and fatigue

Respondents in our survey had a mean PSS score of 14.33 (*SD* ± 6.517; range = 0 to 38). The mean score for the PSWQ was 41.49 (*SD* ± 11.388; range = 16 to 76). Of the total number of respondents, 304 (34%) met the criteria for subclinical levels of anxiety (a minimum score of 45 for non‐clinical samples: Behar, Alcaine, Zuellig, & Borkovec, 2003). Respondents in the study reported a mean CFQ score of 11.98 (*SD* ± 4.69), which is in line with previously reported community data (Cella & Chalder, 2010). The mean for COVID‐19‐specific worries was 3.18 (*SD* ± 1.80; range = 1 to 9.33). The highest level of worry was for the item ‘I am worried that COVID‐19 will result in lasting changes’ mean = 4.20 (*SD* ± 2.97). The lowest levels of worry were for the item ‘I am worried about losing my job’, mean = 2.44 (*SD* ± 2.46). With respect to self‐perceived risk of infection, participants reported a mean likelihood of 32.40% (*SD* ± 27.20) of a COVID‐19 infection by 30 April 2021.

### Association between social connectedness and distress

**Figure 1:**
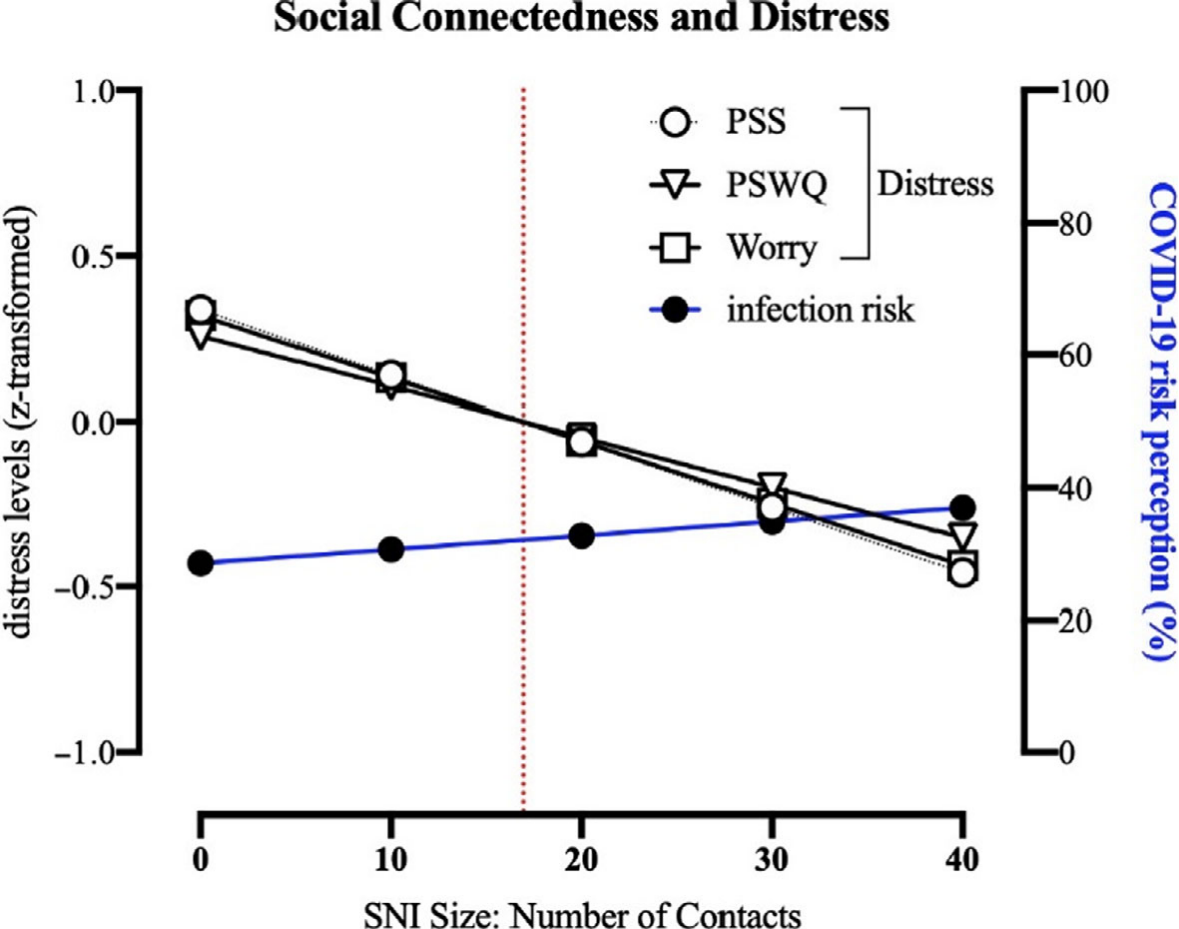
Results of the multiple regression analyses. Left Y‐axis, results for the three z‐standardized‐dependent variables measuring distress (PSWQ, PSS, COVID‐19‐specific worries). Right Y‐axis, results for the dependent variable: perceived likelihood of contracting COVID‐19 within the next 12 months. All analyses included the following covariates: COVID experience, financial worries, age, and gender. The vertical red dotted line indicates the mean values for SNI size. The slopes regressing on distress are significant, whereas the slope regressing on risk perception is not significant.

Models predicting PSS (perceived levels of stress), PSWQ (perceived levels of worries), and COVID‐19‐specific worries showed that social connectedness (as measured by SNI size) was consistently negatively associated with feelings of distress (see Figure 1). All models included age, gender (0 = women; 1 = men), experience with COVID‐19 (0 = no; 1 = yes), and financial worries as covariates.

The model predicting PSS (z‐standardized) was significant (*F*(5,892) = 27.27, *p* < .001, *R* ^2^ = .133), with three significant predictors, SNI size (*b *= −0.020 (SE ± 0.005; 95% CI [−0.029, −0.011]), *t* = −4.336, *p *< .001), age (*b *= −0.016 (SE ± 0.002; 95% CI [−0.021, −0.0118]), *t* = −7.312, *p* < .001), and financial worries (*b *= 0.007 (SE ± 0.001; 95% CI [0.005, 0.009]), *t* = 6.267, *p* < .001). Gender and COVID experience did not predict PSS scores (all *p*s> 0.3).

The model predicting PSWQ (z‐standardized) was also significant (*F*(5,892) = 18.39, *p* < .001, *R* ^2^ = .095), with three significant predictors, SNI size (*b *= −0.015 (SE ± 0.005; 95% CI [−0.024, −0.006), *t* = −3.245, *p* < .001), age (*b *= −0.017 (SE ± 0.002; 95% CI [−0.022, −0.0129]), *t* = −7.667, *p* < .001), and gender (*b *= −0.223 (SE ± 0.065; 95% CI [−0.349, −0.096]), *t* = −3.443 *p* < .001). COVID experience and financial worries were not significantly related to PSWQ scores (all *ps*> 0.2).

The third model, predicting COVID‐19‐specific worries (z‐standardized), was also significant (*F*(4,892) = 25.44, *p* < .001, *R* ^2^ = .1248), with SNI size (*b *= −0.019 (SE ± 0.005; 95% CI [−0.028, −0.010]), age (*b *= −0.013 (SE ± 0.002; 95% CI [−0.017, −0.008]), *t* = −5.613, *p* < .001), and, *t* = −4.102, *p *< .001), gender (*b *= −0.267 (SE ± 0.06; 95% CI [−0.391, −0.142]), *t* = −4.199, *p* < .001), and financial worries (*b *= 0.007 (SE ± 0.001; 95% CI [0.005, 0.009]), *t* = 6.415, *p* < .001) significantly predicting COVID‐19‐specific worries. Experience with COVID‐19 was not significantly associated with COVID‐19‐specific worries (*p* = .72). See Table 1 for an overview of all multiple regression coefficients, and Figure 1 for the effects of age and SNI size on levels of distress and risk perception.

**Table 1:** Unstandardized regression coefficients from models predicting distress, fatigue, and risk perception

	Distress			CFQ Fatigue	Risk perception
	PSS Stress	PSWQ Worry	COVID‐19 Worries		
Age	−.016***	−.017***	−.0125***	−.009***	−0.446***
Gender	−.066	−.223***	−.267***	.038	−2.138
Finance‐worries	.007***	.001	.007***	.005***	0.072*
COVID‐experience	−.033	−.003	.034	.141	7.020**
Social network size	−.020***	−.015**	−.0189***	−.014**	0.211
*R* ^2^	.133***	.094***	.125***	.053***	.083***

Note

The results from five separate regression analyses predicting the following DVs: PSS, PSWQ, COVID‐19 worries, CFQ, and risk perception. The scores for PSS, PSWQ, COVID‐19 Worries, and CFQ were z‐standardized. For all analyses, we entered the following predictor variables: Gender (0 = women; 1 = men); COVID experience (0 = know of no one; 1 = know someone). Social network size = number of individual contacts in the last 2 weeks of lockdown.

* *p* < .05

** *p* < .01

*** *p* < .001.

Next we included the z‐standardized PSWQ scores (general worries) as a covariate in the multiple regression predicting COVID‐19‐specific worries. Here, all previously significant associations remained significant, including SNI size (*b *= −0.013 (SE = 0.004; 95% CI [−0.021, −0.004]), *t* = −2.991, *p* < .001), and age (*b *= −0.005 (SE = 0.002; 95% CI [−0.009, −0.001]), *t* = −2.524, *p* < .001). In addition, the PSWQ score was a significant predictor for COVID‐19‐specific worries (*b *= 0.417 (SE = 0.030; 95% CI [0.358, 0.475]), *t* = 13.980, *p* < .001).

In summary, the results across all three regression models show that distress measures were significantly negatively associated with SNI size indicating that the higher the number of social contacts individuals maintained during the COVID‐19 lockdown, the lower their levels of distress. All models had small to moderate effect sizes, *R* ^2^ = .10–.13 (Cohen, 1992). Furthermore, we found that age was negatively associated with all measures of distress, indicating that the older the respondent, the lower their levels of distress during the COVID‐19 lockdown. In addition, we found that levels of worry (PSWQ and COVID‐19‐specific worries) were lower for men, compared to women. Moreover, financial worries were associated with higher scores on the PSS, as well as greater COVID‐19‐specific worries. For a more in‐depth discussion of the gender‐ and age‐related findings, see the Supplemental Materials.

### Association between social connectedness and risk perception of contracting COVID‐19

Next we predicted the risk perception of getting infected with COVID‐19 within the next year, with SNI size, age, gender, experience with COVID‐19, and financial worries as independent variables. The model was significant (*F*(5,891) = 16.2, *p* < .001, *R* ^2^ = .08335), with three significant predictors: age (*b *= −0.446 (SE = 0.062; 95% CI [−0.567, −0.325]), *t* = −7.233, *p* < .001), experience with COVID‐19 (*b *= 7.020 (SE = 2.286; 95% CI [2.534, 11.507]), *t* = 3.071, *p* = .002), and financial worries (*b *= 0.072 (SE = 0.030; 95% CI [0.013, 0.131]), *t* = 2.394, *p* = .017). Neither gender nor SNI size was associated with infection risk perception (all *p*’s > .1). The knowledge of COVID‐19 cases (of either self or others) was positively associated with risk perception. Similar to all distress measures, higher age was associated with lower risk perception. We do not find an effect of gender on risk perception (see Figure 1).

### Fatigue

We examined the association between SNI size and levels of fatigue (assessed via the CFQ). We first ran a multiple regression controlling for age, gender (0 = women; 1 = men), COVID‐experience, and financial worries. Results showed that the model significantly predicted CFQ scores (*F*(5,892) = 9.983, *p* < .001, *R* ^2^ = .053, z‐transformed). Fatigue was negatively associated with SNI size (*b *= −0.014 (SE = 0.005; 95% CI [−0.024, −0.005]), *t* = −2.993, *p* = .002) and age (*b *= −0.010 (SE = 0.002; 95% CI [−0.014, −0.005]), *t* = −4.061, *p* < .001), but positively associated with financial worries (*b *= 0.005 (SE = 0.001; 95% CI [0.002, 0.007]), *t* = 3.987, *p* < .001). Experience with COVID‐19 and gender was not significantly associated with fatigue (*p *> .10). Results indicate that lower levels of fatigue were associated with higher levels of social connectedness, with a small effect size (*R* ^2^ *= *.053).

**Figure 2:**
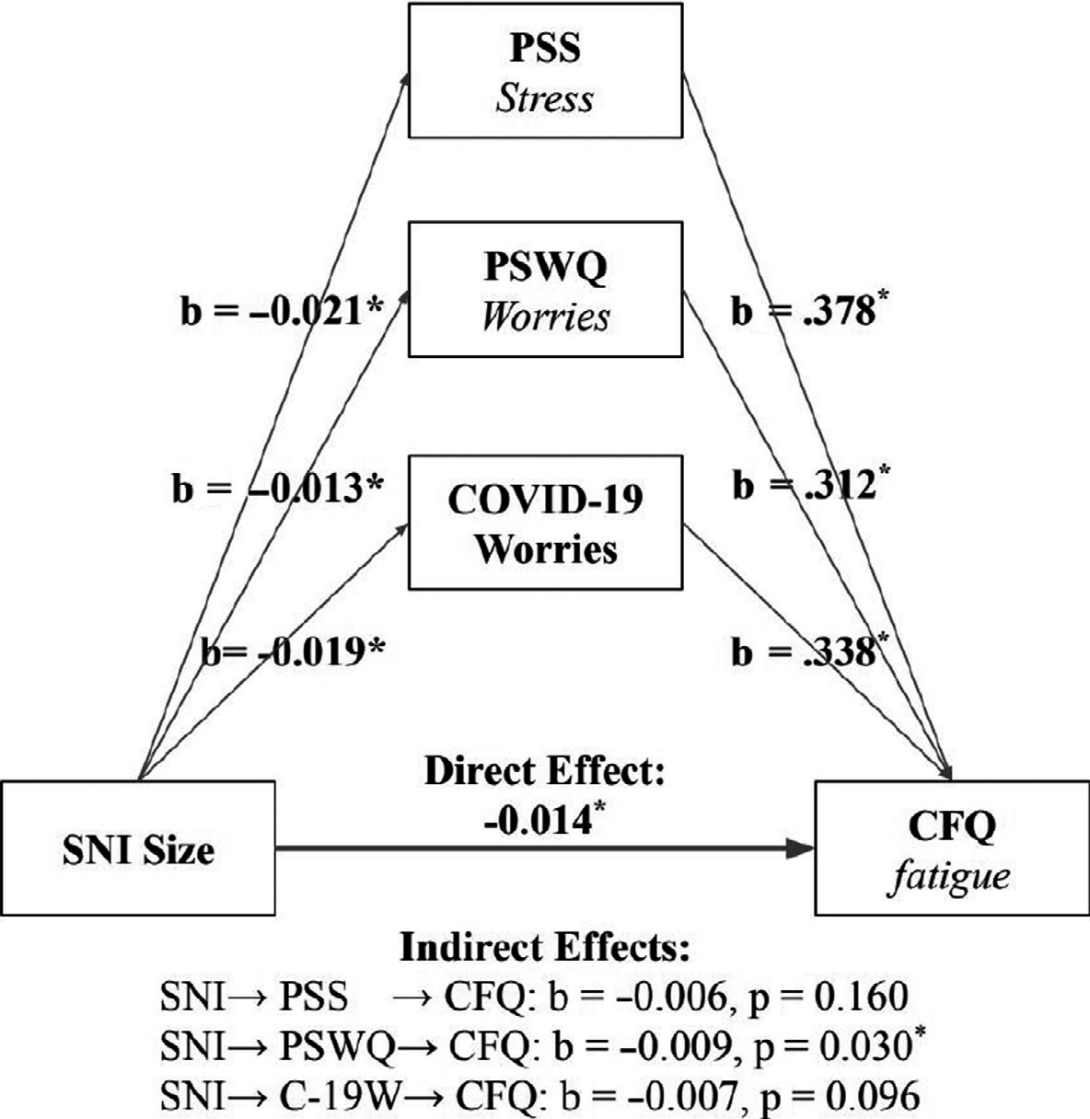
Three independent mediation models explaining the relationship between social network size (SNI size) and CFQ scores (fatigue), mediated by three distress measures (PSS; PSWQ; COVID‐19 worries).These results indicate that social network size can have an indirect effect on somatic symptoms by reducing distress levels.

Next, in order to test the mediating role of distress on the association between SNI size and fatigue, we ran three mediation analyses with the distress measures (PSS, PSWQ, COVID‐19‐specific worries) as mediators (for an overview of the association between variables, see Table S1). First, SNI size was significantly negatively associated with fatigue, *b *= −0.014, *t* = −2.938, *p* < .001. Next, we entered each measure of distress as a mediator, respectively, for each of the three mediations. Measures of distress were negatively associated with SNI size (PSS: *b *= −0.021, *t* = −4.340, *p* < .001; PSWQ: *b *= −0.013, *t* = −2.779, *p* = .005; COVID‐19‐specific worries: *b *= −0.019, *t* = −4.02, *p* < .001*)*, indicating that greater social connectedness was associated with lower levels of distress. Next, the mediation analysis revealed that distress scores were positively associated with fatigue scores (PSS: *b *= 0.378, *t* = 12.122, *p* < .001; PSWQ: *b *= 0.312, *t* = 9.848, *p* < .001; COVID‐19‐specific worries: *b *= 0.338, *t* = 10.681, *p* < .001), indicating that higher levels of distress were associated with higher levels of fatigue. In all three cases, the association between SNI size and fatigue scores was weakened when controlling for levels of distress (PSS: *b *= −0.006, *t* = −1.397, *p* = .16; PSWQ: *b *= −0.009, *t* = −2.168, *p = *.030; COVID‐19‐specific worries: *b *= −0.007, *t* = −1.666, *p* = .096), indicating a mediation of distress on the association between social connectedness and fatigue. The indirect effects of distress on the association between SNI and fatigue were significant for all three measures (PSS: *b *= −0.008, 95% CI [−0.012, −0.004], *p* < .001; PSWQ: *b *= −0.004, 95% CI [−0.00756, −0.001], *p* = .006; COVID‐19 worries: *b *= −0.007, 95% CI [−0.010, −0.003], *p* < .001). Results show that in all three mediation analyses, levels of distress significantly mediated the relationship between network size and fatigue. See Figure 2 for a mediation of COVID‐19 worries on fatigue symptoms.

## Discussion

We investigated the relationship between social connectedness and levels of distress and fatigue in a representative sample in Austria in April 2020. At this time, the country was under lockdown as a result of the COVID‐19 pandemic. This lockdown was comparable to those instituted in other European countries wherein the Austrian government, by means of a governmental decree (Bundesministerium für Soziales, Gesundheit, Pflege und Konsumentenschutz, 2020), required everyone to stay at home, resulting in widespread disruption in people’s normal patterns of social interaction and functioning.

Our results revealed that having a higher number of social contacts in the last two weeks of a nationwide lockdown (i.e., higher social connectedness; Cohen et al., 1997) was associated with lower levels of stress (PSS; Cohen et al., 1994)—and general worry (PSWQ; Meyer et al., 1990), and fatigue (CFQ; Chalder et al., 1993). Moreover, greater social connectedness was also associated with less COVID‐19‐*specific* worries, even when we controlled for general levels of distress. Our data, therefore, support theoretical predictions that increased social connectedness can reduce feelings of distress in general (Holt‐Lunstad, 2018; Snyder‐Mackler et al., 2020), and suggests that increased social connectedness could help to ease people’s anxieties towards a specific traumatic event, such as a global pandemic. Moreover, we demonstrate that worries about the pandemic mediate how social connectedness relates to symptoms of fatigue. We discuss both the theoretical and practical implications of our findings.

### Greater social connectedness is associated with lower levels of stress and worry

Social interactions are associated with improved physical and mental well‐being (Eisenberger & Cole, 2012; Helgeson & Cohen, 1996; Holt‐Lunstad, 2018; Snyder‐Mackler et al., 2020). Epidemiological work has shown that individuals with larger and more active social networks report reduced levels of anxiety (Finch, Okun, Pool, & Ruehlman, 1999; St‐Jean‐Trudel & Guay, 2009) and prospective studies in depression have shown that larger and more diverse social networks predict more favourable clinical outcomes (for additional analyses using the diversity measure of the SNI, see Supplemental Materials) (Santini, Koyanagi, Tyrovolas, Mason, & Haro, 2015; Smith & Christakis, 2008). Our findings add to this literature by demonstrating that, in a representative national sample, greater social connectedness during the COVID‐19 lockdown was associated with lower levels of general stress, and worry.

Larger social networks provide greater opportunities for social support, and this may be one mechanism by which social connectedness could reduce stress and worry. For example, laboratory stress studies have demonstrated that social support can reduce the cortisol response elicited by our body’s stress system—the hypothalamic–pituitary–adrenal (HPA) axis (Heinrichs, Baumgartner, Kirschbaum, & Ehlert, 2003). Moreover, both greater social support and reduced cortisol responses to stress have been associated with reduced activity in brain regions involved in processing threatening situations, such as the dorsal anterior cingulate cortex (Eisenberger, Taylor, Gable, Hilmert, & Lieberman, 2007). This suggests that social support may reduce stress by changing our perceptions of potentially threatening situations (Eisenberger et al., 2007; Morese, Lamm, Bosco, Valentini, & Silani, 2019). However, it is important to note that, although social network size and social support are related (Ashida & Heaney, 2008), several studies have shown that they can also independently predict psychological outcomes (Kroenke et al., 2013; Santini et al., 2015). Increased access to social support is therefore not the sole mechanism by which larger social networks could reduce stress and worry. As such, the number of social connections is just one way to define social connectedness, and importantly, the current study did not obtain measures of perceived quality of social interactions—some individuals will likely benefit from a small number of highly supportive interactions, while others will require more opportunities to connect in order to benefit.

In our sample, participants who reported less social connectedness also reported greater anxieties about the COVID‐19 pandemic, even after controlling for general levels of worry. This supports previous work showing that social network size may be related to more effective coping in response to traumatic events or life changes. For example, social network size was a predictor of psychological resilience following the 9/11 terrorist attacks (Butler et al., 2009). Similarly, women who received a breast cancer diagnosis and had larger social networks were more likely to report a higher quality of life two months post‐diagnosis, even when controlling for levels of social support (Kroenke et al., 2013). These findings demonstrate the fundamental need to promote social contact during, or following traumatic events to help mitigate their potential detrimental psychological impact. Encouragingly, a recent study found that seeking emotional social support has been one of the most common coping strategies to deal with stress during COVID‐19 (Park et al., 2020).

Our findings thus indicate that social connectedness may have played a particularly important role in mitigating distress during the COVID‐19 lockdown. Firstly, people were required to stay at home which potentially resulted in significant disruptions to their normal social lives. Indeed, we found that participants had significantly more contact with others using online communications methods, compared to in‐person interactions. The extent to which non‐personal communication via technology can replace (or compensate for a lack of) face‐to‐face social contact is an important avenue for future work (Vlahovic, Roberts, & Dunbar, 2012). Secondly, the whole population was to some extent affected by the lockdown. Thus, when participants made contact with members of their social networks, there was likely to be increased understanding of their concerns and worries, as people were facing a similar situation (Singer & Lamm, 2009). This may have been especially important in reducing participants’ COVID‐19‐specific worries—in the same way that peer support groups for certain diseases, such as cancer, may be particularly beneficial in reducing distress related to the diagnosis (Ussher, Kirsten, Butow, & Sandoval, 2006). Together, these findings underline the importance of fostering and maintaining social connections, particularly during times of adversity, in order to reduce stress and worry (Vinkers et al., 2020).

### Distress mediates the association between social connectedness and fatigue

Our results also demonstrate that the negative relationship between fatigue and social connectedness is mediated by feelings of distress (stress, general worries, and COVID‐19‐specific worries—respectively), suggesting that social connectedness might be a resilience factor for somatic symptoms associated with distress. 'Specifically we found that those with smaller network sizes, who were highly distressed during the pandemic, were also likely to feel more fatigued. This, in turn, might result in a reduced activity, including engaging in social contact with fewer people. These associations have been suggested in public health data previously in very specific samples, for example, how fatigue and stress intersect in healthcare workers, teachers, or prison staff (Burke, Greenglass, & Schwarzer, 1996; Cho et al., 2019; Keinan & Malach‐Pines, 2007), but never in a representative sample that is in its entirety impacted by a common stressor. The findings support a view that although fatigue can have different sources including inflammation, physical exertion, mental exertion, and stress, it has a common consequence—a reduction in levels of activity (Afari & Buchwald, 2003; Draper et al., 2018; Hockey & Hockey, 2013; Müller & Apps, 2019). Our findings point to a partially psychological foundation of fatigue that can be mediated by specific worries and is linked to social connectedness. Theories suggest that such factors may interact with people’s levels of motivation, with higher levels of fatigue reducing the willingness to engage in subsequent activities.

### Limitations and future directions

Our results should be considered alongside their limitations and considerations for future research. First, these data were collected during lockdown and we do not have pre‐lockdown baseline assessments when physical distancing measures were not in place. Thus, it could be argued that our findings only apply to the time‐period when most of the population was required to stay at home. However, our key finding that social connectedness was associated with reduced distress has been consistently demonstrated in a range of studies outside of pandemic and government imposed lockdown situations (Eisenberger & Cole, 2012; Helgeson & Cohen, 1996; Holt‐Lunstad, 2018; Snyder‐Mackler et al., 2020). Moreover, examining this association during a time of crisis has allowed us to test the robustness of these experimental associations in a real‐life situation. The absence of a suitable baseline period may also negate the specific conclusions we can draw about social connectedness and distress during the lockdown. However, we found that social connectedness was associated with COVID‐19‐specific worries even when we controlled for general levels of worry. This suggests that social connectedness may help us deal with the impact of specific traumatic events, such as a global pandemic or a terrorist attack (Butler et al., 2009), rather than simply being related to levels of distress in general.

Secondly, our data cannot determine the nature of the association between distress and social connectedness. It is likely that this relationship is bidirectional—social connectedness could protect against increased distress (Santini et al., 2015; Smith & Christakis, 2008), especially during times of widespread social disruption. Conversely, those who report higher levels of distress may also be less likely to seek social contact with others (Matthews & Tye, 2019). Prospective studies are needed to disentangle the directionality of these effects.

Thirdly, although we found an association between social connectedness and reduced distress, it is unclear *how* a greater number of social connections may result in reduced distress. For example, having a greater number of social connections may result in more frequent social interactions, easier access to social support, and a greater sense of belonging (Wills & Shinar, 2000). Yet, while these factors are related, they are likely to play distinct roles when dealing with traumatic events. For example, it has been shown that the diversity of the social network may be more protective against post‐traumatic stress disorder than perceptions of social support (Platt, Keyes, & Koenen, 2014). Moreover, we were able to show through mediation analyses that levels of distress (i.e., PSS, PSWQ, COVID‐19‐specific worries) mediate the association between social network size and fatigue, providing more detail on how the number of connections we have impacts feelings of exhaustion that profoundly affect everyday functioning. More broadly, establishing which factors best account for the association between social connectedness and reduced distress, particularly during periods of social and economic disruption, is an important aim for future work.

Fourthly, we recruited participants in the last week of lockdown in Austria. Given the unpredictability of the crisis, during the survey preparation and deployment we were not aware that the lockdown would end at the end of April. We speculate that if this survey would have been deployed at an earlier time‐point during the lockdown we would have observed higher levels of distress (as reported in data from the UK; Fancourt, Bu, Mak, & Steptoe, 2020), and possibly lower levels of social connectedness (due to people adjusting to the lockdown measures). As such, we would predict an even stronger association in the first weeks, when uncertainty was higher. Future studies, such as if second‐wave lockdowns occur, could test this hypothesis.

Finally, we did not obtain clinical measurements of psychopathologies, such as anxiety disorders or depression. Given the immediacy of the COVID‐19 crisis, and the relatively short time frame in which the situation unfolded, measures of worry and stress were more appropriate to test the short‐term impact on mental health. As such, the assessment of long‐term health outcomes was beyond the scope of this investigation. Future research will need to investigate this further, notably with a longer time frame from the onset of the pandemic. It is worth noting, however, that the association between social connectedness and worry was also found in those participants who scored at the subclinical end of the PSWQ (see Supplemental Materials), suggesting that our findings could extend to clinical samples.

### Conclusion

We collected data from a representative sample of Austrian citizens experiencing lockdown as a result of the COVID‐19 pandemic. We showed that individuals who had had contact with a greater number of people in the previous two weeks (i.e., had high levels of social connectedness) reported lower levels of stress, worry, and fatigue. Social connectedness was also inversely associated with more worry related specifically to the COVID‐19 pandemic even after controlling for participants’ general worry. Finally, we found that levels of distress mediated the association between social connectedness and fatigue. Overall, we demonstrated that increased social connectedness during difficult and uncertain times can buffer against negative physical and mental health outcomes, and promote resilience. These results, therefore, highlight the importance of fostering and maintaining social connections during adversity.

## Authors’ contributions

JPN and CL developed the study concept and design. JPN conducted the data processing and data analyses. All authors contributed to the interpretation of the data. JPN drafted the manuscript with the help of PAGF and NA. JC, PLL, MAJA, and CL provided critical revisions. All authors approved the final version of the manuscript for submission.

## Funding

This study was supported by a COVID‐19 Rapid Response grant from the University of Vienna (to CL), and the Austrian Science Fund (FWF, I3381, to CL). JPN holds a doctoral scholarship from Fonds de Recherche du Québec–Société et Culture (FRQSC).

## Conflicts of interest

The authors have no financial or other conflicts of interest to declare.

## Supporting information


**Data S1.** Discussion: age and gender effects.
**Data S2.** Mediation analysis ‐ Age, COVID‐19 specific worries, and financial worries.
**Data S3.** Social network index ‐ Diversity scores.
**Data S4.** High worry sub‐sample analyses.
**Table S1.** Correlations.

## Data Availability

Data are made available via the OSF platform (see osf.io/v8pjn/).

## Footnotes

^1^ Stay‐at‐home measures meant that individuals in Austria were discouraged from leaving their apartments and houses, and in‐person contact was limited to the people in the same household. Apart from essential businesses (supermarkets, pharmacies, and drug stores), all businesses were closed as was access to most public parks and playgrounds. Schools and (in most cases) child day‐care centres were closed (Bundesministerium für Soziales, Gesundheit, Pflege und Konsumentenschutz, 2020).

^2^ Note, all subsequent analyses yield comparable results when including all previously excluded participants. All models and all predictors remain significant.

^3^ Running these regression analyses without the covariates (gender, age, COVID‐experience, financial worries) did not change the significance of the association between social network size and measures of distress.
