# Supplementary material for: Resilience during uncertainty? Greater social connectedness during COVID‐19 lockdown is associated with reduced distress and fatigue
Source: Br J Health Psychol. 2020 Oct 25;26(2):553–69. doi: 10.1111/bjhp.12485 (PMC8247344; doi:10.1111/bjhp.12485)
Supplement: Supplementary file 1 — Data S1. Discussion: age and gender effects. Data S2. Mediation analysis ‐ Age, COVID‐19 specific worries, and financial worries. Data S3. Social network index ‐ Diversity scores. Data S4. High worry sub‐sample analyses. Table S1. Correlations. [file BJHP-26-553-s001.docx]

**Supplemental Materials for:**

Resilience During Uncertainty? Greater Social Connectedness During COVID-19 Lockdown is Associated with Reduced Distress and Fatigue.

Jonas P. Nitschke^1,2*^, Paul A.G. Forbes^1^, Nida Ali^3^, Jo Cutler^4,5^, Matthew A.J. Apps^4,5^, Patricia L. Lockwood^4,5^, Claus Lamm^1*^

1 Department for Cognition, Emotion, and Methods in Psychology, University of Vienna, Vienna, Austria

2 Department of Psychology, McGill University, Montréal, Canada

3 Department of Psychology, Clinical and Health Psychology, University of Vienna, Vienna, Austria

4 Department of Experimental Psychology, University of Oxford, Oxford, United Kingdom

5 Centre for Human Brain Health, School of Psychology, University of Birmingham, United Kingdom

**This document includes**:

Supplemental Analyses

1. Discussion: Age and Gender Effects
2. Mediation analysis - Age, COVID-19 specific worries, and financial worries
3. Social Network Index - Diversity Scores
4. High worry sub-sample analyses
5. **Table S1.** Correlations
6. **Discussion: Age and Gender Effects**

*Younger participants and women reported greater levels of distress*

Younger participants reported higher levels of stress, general worry, COVID-19 specific worry, and a higher perceived likelihood of getting infected within the next year, compared to older participants. These effects remained even after controlling for financial concerns related to the COVID-19 pandemic. Moreover, women, compared to men, reported higher levels of worries, both general and COVID-19 specific worries. Indeed, higher levels of worry as observed in women, and younger participants in our study, is in line with previous work using the PSWQ and other measures of worry and anxiety which show robust age and gender effects [(Crittendon & Hopko, 2006; McCann et al., 1991; Wittchen & Hoyer, 2001)](https://paperpile.com/c/a3yyOv/7UuWM+5BHBJ+SzAmj). In addition, we found that gender and age were also related to worries specific to COVID-19, even when controlling for general levels of worry. Older age is one of the greatest risk factors for disease severity and death from COVID-19 [(Jordan et al., 2020)](https://paperpile.com/c/a3yyOv/nJZYg). Similarly, mortality rates are higher for men compared to women [(Wenham et al., 2020)](https://paperpile.com/c/a3yyOv/KoYGu). At first glance, it seems somewhat counterintuitive - we found increased worry in those least at risk of death and serious disease. However, the risk of death and serious disease at a population level were relatively low in Austria where infection rates and mortality rates were lower in comparison to other European countries, Canada, or the United States (<https://covid19.who.int/>), and the health-care system was not overburdened or at risk of collapsing as in other countries, such as Italy or Spain. Thus, in our study, women and younger participants may have been more affected by the wider social and economic implications of the pandemic. For example, greater financial worries were associated with younger age in our sample and this partially mediated the effect of age on COVID-19 worries (see point below). This fits with data from other European countries showing that younger people, especially those aged 18-24 years old, were more likely to have their employment affected by the pandemic, either through losing their job or having their hours reduced ([Resolution Foundation, 2020](https://www.resolutionfoundation.org/app/uploads/2020/05/Young-workers-in-the-coronavirus-crisis.pdf)). Similarly, closure of communal social spaces, such as parks, bars, restaurants, sport facilities, schools, and universities, may have had a greater impact on the lives of younger participants. We did not find evidence for gender differences for financial worries. However, it could be that women were more affected by the lockdown in other ways. For example, women may have been more likely to carry the burden of increased household and childcare responsibilities due to the closure of schools and kindergartens [(Wenham et al., 2020)](https://paperpile.com/c/a3yyOv/KoYGu). Future studies should systematically investigate factors influencing these age and gender differences in COVID-19 related worry to test these possibilities. This will help us to better protect those most affected by the current crisis and any future pandemics.


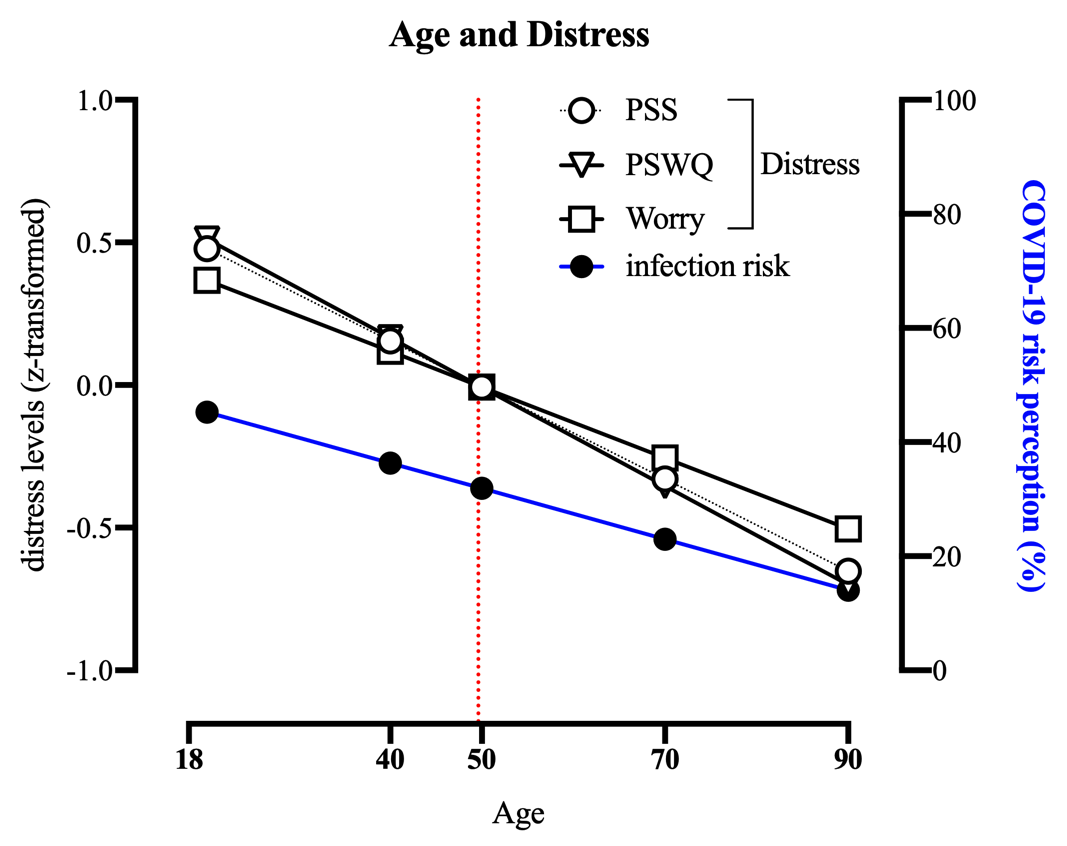


**Figure S1.** Results of the multiple regression analyses. Left Y-Axis, results for the three z-standardized dependent variables measuring distress (PSWQ, PSS, COVID-19 specific worries). Right Y-Axis, results for the dependent variable: perceived likelihood of contracting COVID-19 within the next 12 months. All analyses included the following covariates: COVID-experience, financial worries, age, and gender. The vertical red dotted line indicates the mean age. All slopes are significant (see Table 1).

1. **Mediation analysis - Age, COVID-19 specific worries, and financial worries**

To test if financial worries could partially explain the effect of age on COVID-19 related worries we ran a mediation analysis [(Baron & Kenny, 1986)](https://paperpile.com/c/a3yyOv/rzOi) with COVID-worries as dependent variable, age as independent variable, and financial worries as mediator. The Analysis was conducted using R [(R Core Team, 2020)](https://paperpile.com/c/a3yyOv/Quod) and the ‘mediation’ package [(Tingley et al., 2014)](https://paperpile.com/c/a3yyOv/D0lu).

Results of the mediation analysis showed a significant (partial) mediation. The effect of age on COVID-related worries was partially mediated by the amount of financial worries related to COVID-19. As Figure S1 illustrates, the regression coefficient between financial worries and COVID-10 related worries was significant (b= 0.212, *t*= 6.580, *p*< 0.01). The indirect effect was (-0.011 * 0.212)= -0.00239. We tested the significance of this indirect effect using bootstrapping procedures. Unstandardized indirect effects were computed for each of 1000 bootstrapped samples, and the 95% confidence interval was computed by determining the indirect effects at the 2.5th and 97.5th percentiles. The bootstrapped unstandardized indirect effect was -0.00239, and the 95% confidence interval ranged from -0.00367 to 0. The indirect effect was statistically significant (*p*< 0.001).


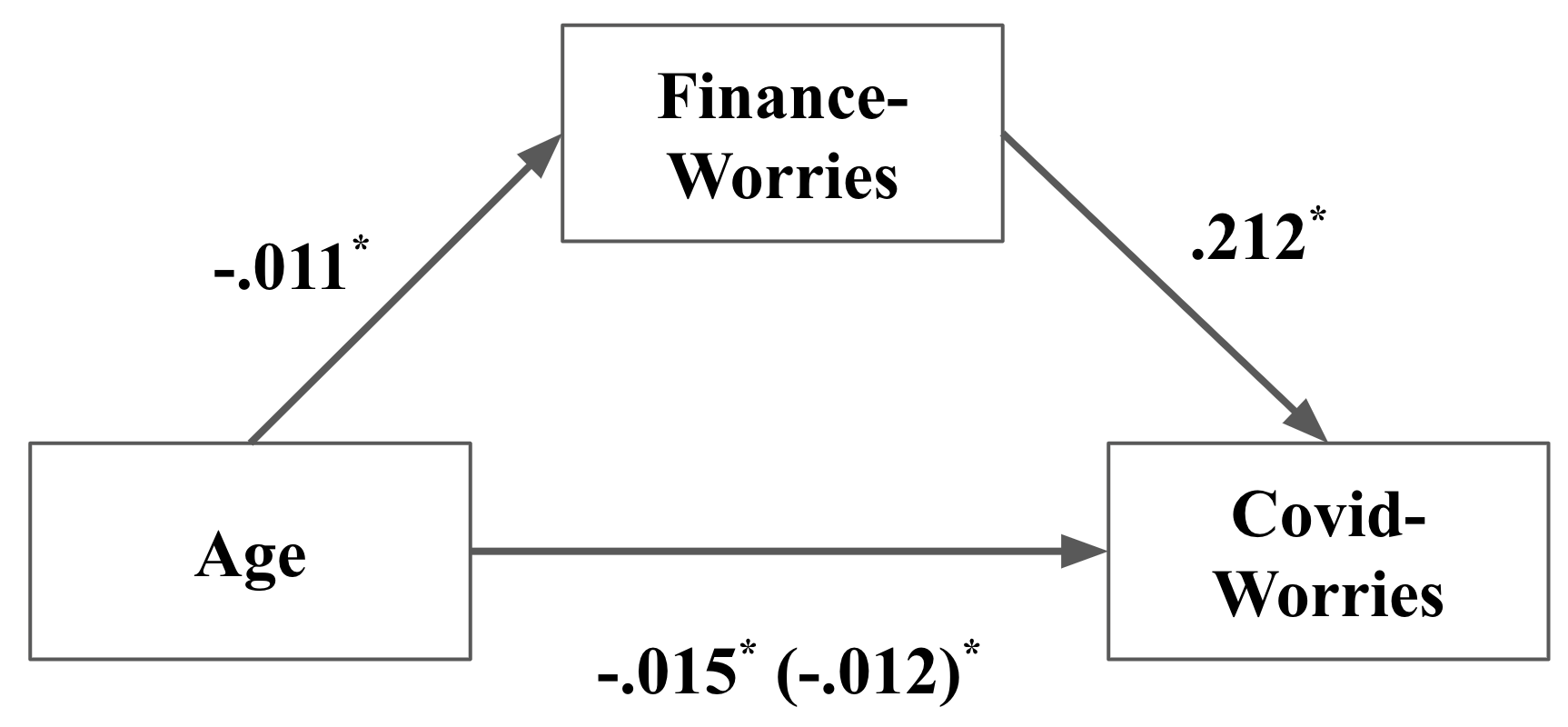


**Figure S2:** Mediation Analysis. Financial worries partially mediated the relationship between age and COVID-worries. Older individuals had lower levels of financial worries, which partially explained lower COVID-19 related worries for older individuals.

1. **Social Network Index - Diversity Scores**

In addition to social network size (i.e., numbers of unique connections), we used the SNI to derive a measure of Social Network Diversity (the number of social roles participants regularly engaged in). Social Network Diversity refers to the number of high contact social roles a respondent engages in [(Cohen & Lemay, 2007)](https://paperpile.com/c/a3yyOv/Brjm). High contact roles are defined as those in which the respondent reports engaging in at least once every two weeks. Higher network Diversity has previously been associated with better health practices and well-being [(Cohen & Lemay, 2007)](https://paperpile.com/c/a3yyOv/Brjm). SNI size and SNI diversity were significantly correlated, *r*= 0.7710771, *p*< 0.001.

We ran three independent multiple regressions (cf. main manuscript) predicting measures of distress (PSS, PSWQ, COVID-Worries), with age, gender (0=women; 1=men), COVID-experience (0=no; 1=yes), financial worries, and the SNI diversity score. The results show that in all three multiple regressions SNI diversity was significantly associated with the dependent variable. For the PSS: b=-0.057, *t*= -3.533, *p*< 0.001; for the PSWQ: b= -0.033, *t*= -2.022 , *p*= 0.04; for COVID-Worries: b= -0.050, *t*= -3.074, *p*= 0.002.

Similar to the results using the SNI size (i.e, number of unique social connections), SNI diversity (i.e., number of high social roles) was significantly negatively associated with measures of distress.

1. **High worry subsample analyses**

To test the association between worry and social network size we used the Penn State Worry Questionnaire [(PSWQ; Meyer et al., 1990)](https://paperpile.com/c/a3yyOv/NXFp/?prefix=PSWQ%3B). Of the total number of respondents, 304 (34%) met the criteria for high levels of worry, possibly indicating subclinical levels of anxiety [(a minimum score of 45 for non-clinical samples: Behar et al., 2003)](https://paperpile.com/c/a3yyOv/Jexe/?prefix=a%20minimum%20score%20of%2045%20for%20non-clinical%20samples%3A). Here, we set out to test the association between Worry (PSWQ) and SNI size in this subsample of participants. We ran the same regression analysis described in the main manuscript, but only in participants that met the cut-off criteria of 45 [(Behar et al., 2003)](https://paperpile.com/c/a3yyOv/Jexe). Age, financial worries, COVID-experience (0=no; 1=yes), and gender (0=women; 1=men) were included as covariates.

The model was significant (*F*(5, 296 )= 7.802 *p*< 0.001, *R*^2^ =0.1164), with two significant predictors, age (b= -0.017, se= 0.004, *t*= -4.579, *p*< 0.001), and SNI size (b= -0.0291, se= 0.008, *t*= -3.476, *p*< 0.001). Gender, financial worries, and covid-19 experience were not associated with levels of general worry (PSWQ).

Similarly, when predicting COVID-19 specific worries in the subsample of highly worried individuals (as measured by the PSWQ), the model was significant (*F*(5, 296)= 10.61, *p*< 0.001, *R*^2^= 0.152), with three significant predictors; gender (b= -0.442, se= 0.112, *t*= -3.968, *p*< 0.001), financial worries (b= 0.009, se= 0.002, *t*= 5.472, *p*< 0.001), and SNI size (b= -0.017, se= 0.008, *t*= -2.169 , *p*< 0.03).

**Table S1.** Correlations

*Means, standard deviations, and correlations with confidence intervals*

| *Variable* | *M* | *SD* | *1* | *2* | *3* | *4* | *5* | *6* | *7* |
| --- | --- | --- | --- | --- | --- | --- | --- | --- | --- |
|  |  |  |  |  |  |  |  |  |  |
| 1. Age | 49.57 | 14.45 |  |  |  |  |  |  |  |
|  |  |  |  |  |  |  |  |  |  |
| 2. PSS | 14.31 | 6.52 | -.26** |  |  |  |  |  |  |
|  |  |  | [-.32, -.20] |  |  |  |  |  |  |
|  |  |  |  |  |  |  |  |  |  |
| 3. PSWQ | 41.41 | 11.44 | -.26** | .54** |  |  |  |  |  |
|  |  |  | [-.32, -.20] | [.49, .58] |  |  |  |  |  |
|  |  |  |  |  |  |  |  |  |  |
| 4. COVID-19 Worries | 18.84 | 10.82 | -.22** | .53** | .47** |  |  |  |  |
|  |  |  | [-.28, -.16] | [.48, .58] | [.42, .52] |  |  |  |  |
|  |  |  |  |  |  |  |  |  |  |
| 5. CFQ | 11.98 | 4.69 | -.15** | .38** | .32** | .34** |  |  |  |
|  |  |  | [-.21, -.08] | [.33, .44] | [.26, .38] | [.29, .40] |  |  |  |
|  |  |  |  |  |  |  |  |  |  |
| 6. Risk perception | 32.07 | 27.14 | -.25** | .12** | .12** | .11** | .10** |  |  |
|  |  |  | [-.31, -.19] | [.05, .18] | [.05, .18] | [.04, .17] | [.04, .17] |  |  |
|  |  |  |  |  |  |  |  |  |  |
| 7. Financial worries | 20.60 | 29.55 | -.16** | .25** | .09** | .24** | .16** | .11** |  |
|  |  |  | [-.23, -.10] | [.19, .31] | [.02, .15] | [.18, .30] | [.10, .23] | [.04, .17] |  |
|  |  |  |  |  |  |  |  |  |  |
| 8. SNI Size | 16.91 | 6.90 | -.05 | -.14** | -.09** | -.13** | -.10** | .07* | -.09** |
|  |  |  | [-.12, .01] | [-.21, -.08] | [-.16, -.03] | [-.20, -.07] | [-.16, -.03] | [.01, .14] | [-.15, -.02] |
|  |  |  |  |  |  |  |  |  |  |

*Note.*PSS: Perceived Stress Scale; PSWQ: Penn-State Worry Questionnaire; COVID-19 Worries: COVID-19 related worries; Risk perception: Risk perception of contracting COVID-19 within the next year. Financial worries: Worries about financial future. M and SD are used to represent mean and standard deviation, respectively. *M* and *SD* are used to represent mean and standard deviation, respectively. Values in square brackets indicate the 95% confidence interval for each correlation. The confidence interval is a plausible range of population correlations that could have caused the sample correlation [(Cumming, 2014)](https://paperpile.com/c/a3yyOv/iWBx). All correlations are uncorrected. * indicates *p* < .05. ** indicates *p* < .01.

**Additional References**

[Baron, R. M., & Kenny, D. A. (1986). The moderator–mediator variable distinction in social psychological research: Conceptual, strategic, and statistical considerations. *Journal of Personality and Social Psychology*, *51*(6), 1173–1182.](http://paperpile.com/b/a3yyOv/rzOi)

[Behar, E., Alcaine, O., Zuellig, A. R., & Borkovec, T. D. (2003). Screening for generalized anxiety disorder using the Penn State Worry Questionnaire: a receiver operating characteristic analysis. *Journal of Behavior Therapy and Experimental Psychiatry*, *34*(1), 25–43.](http://paperpile.com/b/a3yyOv/Jexe)

[Cohen, S., & Lemay, E. P. (2007). Why would social networks be linked to affect and health practices? *Health Psychology: Official Journal of the Division of Health Psychology, American Psychological Association*, *26*(4), 410–417.](http://paperpile.com/b/a3yyOv/Brjm)

[Crittendon, J., & Hopko, D. R. (2006). Assessing worry in older and younger adults: Psychometric properties of an abbreviated Penn State Worry Questionnaire (PSWQ-A). *Journal of Anxiety Disorders*, *20*(8), 1036–1054.](http://paperpile.com/b/a3yyOv/7UuWM)

[Cumming, G. (2014). The new statistics: why and how. *Psychological Science*, *25*(1), 7–29.](http://paperpile.com/b/a3yyOv/iWBx)

[Jordan, R. E., Adab, P., & Cheng, K. K. (2020). Covid-19: risk factors for severe disease and death [Review of *Covid-19: risk factors for severe disease and death*]. *BMJ* , *368*, m1198.](http://paperpile.com/b/a3yyOv/nJZYg)

[McCann, S. J., Stewin, L. L., & Short, R. H. (1991). Sex differences, social desirability, masculinity, and the tendency to worry. *The Journal of Genetic Psychology*, *152*(3), 295–301.](http://paperpile.com/b/a3yyOv/SzAmj)

[Meyer, T. J., Miller, M. L., Metzger, R. L., & Borkovec, T. D. (1990). Development and validation of the Penn State Worry Questionnaire. *Behaviour Research and Therapy*, *28*(6), 487–495.](http://paperpile.com/b/a3yyOv/NXFp)

[R Core Team. (2020). *R: A Language and Environment for Statistical Computing*. R Foundation for Statistical Computing.](http://paperpile.com/b/a3yyOv/Quod) [https://www.R-project.org/](https://www.r-project.org/)

[Tingley, D., Yamamoto, T., Hirose, K., Keele, L., & Imai, K. (2014). *mediation: R package for causal mediation analysis*.](http://paperpile.com/b/a3yyOv/D0lu) <https://dspace.mit.edu/handle/1721.1/91154?show=full>

[Wenham, C., Smith, J., Morgan, R., & Gender and COVID-19 Working Group. (2020). COVID-19: the gendered impacts of the outbreak. *The Lancet*, *395*(10227), 846–848.](http://paperpile.com/b/a3yyOv/KoYGu)

[Wittchen, H. U., & Hoyer, J. (2001). Generalized anxiety disorder: nature and course. *The Journal of Clinical Psychiatry*, *62 Suppl 11*, 15–19; discussion 20–21.](http://paperpile.com/b/a3yyOv/5BHBJ)
